# A Simple Method for Synthesis of Chitosan Nanoparticles with Ionic Gelation and Homogenization

**DOI:** 10.3390/molecules28114328

**Published:** 2023-05-25

**Authors:** Nicolas Van Bavel, Travis Issler, Liping Pang, Max Anikovskiy, Elmar J. Prenner

**Affiliations:** 1 Department of Biological Sciences, University of Calgary, Calgary, AB T2N 1N4, Canada; nicolas.vanbavel@ucalgary.ca (N.V.B.); travis.issler@ucalgary.ca (T.I.); 2 Institute of Environmental Science and Research, P.O. Box 29181, Christchurch 8540, New Zealand; 3 Department of Chemistry, University of Calgary, Calgary, AB T2N 1N4, Canada

**Keywords:** nanoparticle synthesis, chitosan, biopolymers, ionic gelation

## Abstract

Chitosan nanoparticles (CNPs) are known to have great utility in many fields (pharmaceutical, agricultural, food industry, wastewater treatment, etc.). In this study we aimed to synthesize sub-100 nm CNPs as a precursor of new biopolymer-based virus surrogates for water applications. We present a simple yet efficient synthesis procedure for obtaining high yield, monodisperse CNPs with size 68–77 nm. The CNPs were synthesized by ionic gelation using low molecular weight chitosan (deacetylation 75–85%) and tripolyphosphate as crosslinker, under rigorous homogenization to decrease size and increase uniformity, and purified by passing through 0.1 μm polyethersulfone syringe filters. The CNPs were characterized using dynamic light scattering, tunable resistive pulse sensing, and scanning electron microscopy. We demonstrate reproducibility of this method at two separate facilities. The effects of pH, ionic strength and three different purification methods on the size and polydispersity of CNP formation were examined. Larger CNPs (95–219) were produced under ionic strength and pH controls, and when purified using ultracentrifugation or size exclusion chromatography. Smaller CNPs (68–77 nm) were formulated using homogenization and filtration, and could readily interact with negatively charge proteins and DNA, making them an ideal precursor for the development of DNA-labelled, protein-coated virus surrogates for environmental water applications.

## 1. Introduction

**Figure 1:**
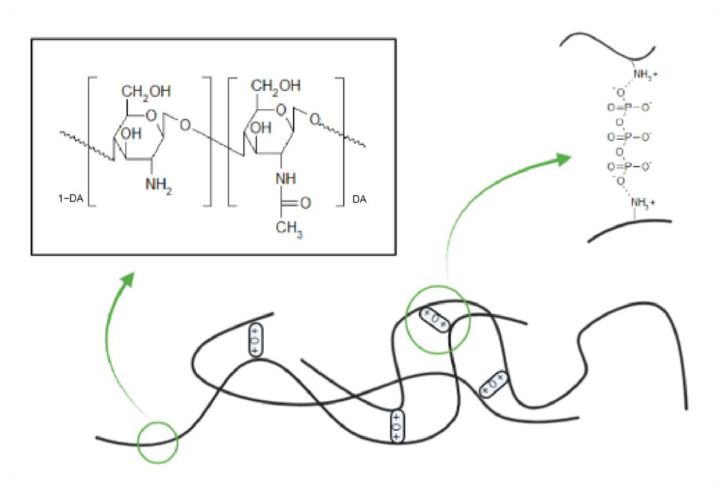
Chitosan crosslinking with tripolyphosphate, showcasing the structure of chitosan polymers and the anionic crosslinker tripolyphosphate. Created with BioRender.com.

Chitosan is an abundant natural cationic biopolymer derived from the partial deacetylation of chitin [1]. The biopolymer is made up of linear β-(1→4) glycosidic linkages of 2-acetamido-d-glucose and 2-amino-d-glucose units (Figure 1). The amine functional groups of chitosan have a pKb value of ~6.5, which increases its solubility in neutral and acidic media [2]. Additionally, the amine groups contribute to the physiochemical properties of the biopolymer and allow for chitosan-based materials to be developed via synthetic processes such as gelation.

**Figure 2:**
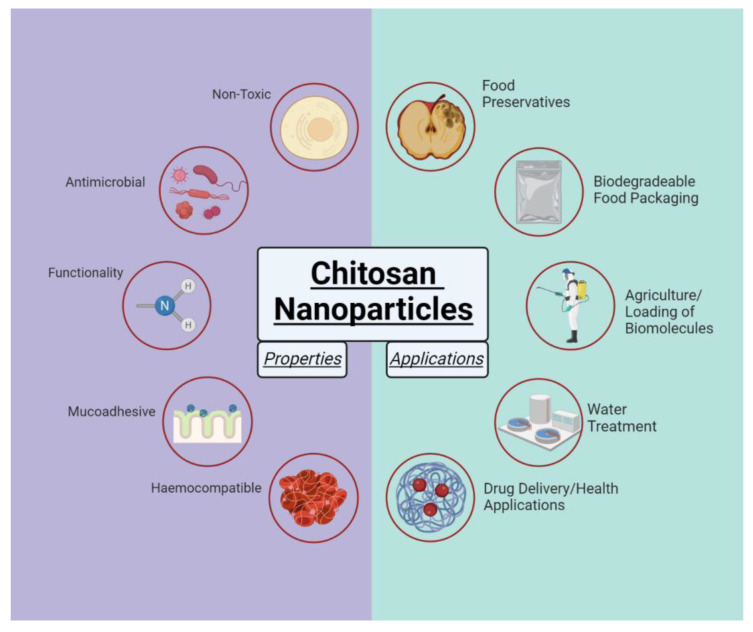
Properties and applications of chitosan nanoparticles. Adapted from “Increased Risk of Cancers with Obesity.” Created with BioRender.com.

Chitosan is biodegradable, biocompatible, nontoxic and has notable antimicrobial properties. Chitosan nanoparticles (CNPs), microparticles (CMPs) and membranes have attracted considerable interests in applications in food industry, water and wastewater treatment, drug delivery and pharmaceutical industry [3,4,5,6,7] (Figure 2).

Chitosan-based materials have been used as antimicrobial agents in the food industry as a food preservative [8]. Chitosan-coated foods were found to substantially reduce surface microbial colonies [9]. Moreover, the application of CNPs to reinforce the biodegradable thermoplastic polylactic acid may become a key method in the production of biodegradable food packaging [10,11]. The antimicrobial property of chitosan is largely attributed to its amine groups, which enable the polymer to bind the negatively charged bacterial cell walls, ultimately leading to changes in membrane permeability [12,13]. Chitosan disrupts biofilm formation, is mucoadhesive and capable of opening tight junctions in epithelial cells [14,15,16,17].

CNPs have demonstrated great utility as a drug delivery vehicle, acting as scaffolding for the loading of drugs and other biomolecules. Specifically, CNPs have been reported to improve drug release, permeability, and stability [18]. Furthermore, the ability for CNPs to be functionalized with specific markers may allow for targeted delivery with a range of therapeutics. For its pharmaceutical uses, chitosan has been implemented to enhance the uptake of beneficial chemical compounds, such as flavonoids. For example, incorporation of tea polyphenols into chitosan nanoparticles has been shown to increase the absorption and bioavailability of the phenols [19]. 

Applications in the agricultural industry include herbicide-loaded CNPs as a more environmentally friendly option compared to traditional weed control methods [20], which may reduce the impact of herbicides on the environmental and human health.

In the field of wastewater treatment, the hydroxyl groups associated with chitosan make the biopolymer a promising candidate for the removal of pesticides, dyes, and heavy metals. Magnetic CNPs prepared with iron oxide (Fe_3_O_4_) have been demonstrated as an effective tool for removing heavy metals and dyes from wastewater [21,22].

CNPs are routinely formulated with the ionic gelation method, in which polymer units of interest are cross-linked through electrostatic interactions with an oppositely charged molecule [23]. For CNP synthesis, an anionic cross linker such as tripolyphosphate (TPP) may be used (Figure 1). While setup and execution of this synthesis method is relatively simple, the desired outcome with a target size and uniformity may not be so readily achieved. Synthesis parameters including homogenization time and speed must be carefully tuned, while parameters such as pH and ionic strength may need to be considered to avoid aggregation and excess growth of the particles [24,25].

For most applications, it is essential to control the size and size distribution of NPs, which play a critical role in determining material properties and performance [26,27,28]. Small CNPs (30–60 nm) are highly efficient for DNA encapsulation and are an effective vehicle for delivery of DNA vaccines [29]. 

For water and wastewater treatment, smaller particles will allow for a greater degree of adsorption thus the large surface area-volume ratio of CNPs is of great interest. The formulation of sub-100 nm CNPs with a high degree of uniformity is particularly applicable in water and wastewater treatment as a potential surrogate for waterborne viruses. In this way, CNPs may be labelled with a marker, and used to determine the fate of viruses within a treatment facility. 

Previously, protein-coated, DNA-labelled 70 nm silica nanoparticles have been used as virus surrogates for investigating virus removal and transport in groundwater and soils [30,31,32]. However, silica NPs-based virus surrogates are neither biocompatible nor biodegradable thus have limited uses in the nature environments. To address a lack of appropriate virus substitutes made of biocompatible biopolymers, we aimed to produce uniform sub-100 nm CNPs as a precursor for the development of biopolymer-based virus surrogates. The effects of pH, ionic strength and purification methods on the size and polydispersity of CNPs formation during ionic gelation were examined. Finally, future work was discussed. 

## 2. Results and Discussion

### 2.1. Chitosan Nanoparticle Synthesis

In this study we successfully synthesized high yield (3.57 × 10^9^ particles/mL) monodispersed (PDI 0.2–0.3) CNPs with size 68–77 nm using the ionic gelation method. This size range is very similar to some enteric viruses for example rotavirus and adenovirus. Thus, the produced CNPs can be used as a precursor for developing novel biopolymer-based virus surrogates, which will be DNA-labelled and protein-coated to mimic the physicochemical properties of the target viruses [30]. The new virus surrogates can be used to predict water contamination risks in freshwaters and help to design improved water treatment systems. Such a biopolymer-based abiotic virus surrogate has not been reported in the literature.

The virus-sized CNPs were obtained under the following optimized conditions: initial concentrations: 1 mg/mL (final 0.7 mg/mL) LMW chitosan (50–190 kDa, deacetylation 75–85%) (pH 3.2), 1 mg/mL TPP (final 0.3 mg/mL). Samples were homogenized using a handheld homogenizer motor (7000 rpm for 2 min), and purified by passing through a 0.1 µm syringe filter at flow rate 0.15 mL/min. Larger CNPs (95–219 nm) were produced under TPP pH 2–4, NaCl 100–200 mM, and when using ultracentrifugation and size exclusion chromatography. Particles reached >1000 nm at 200 mM NaCl.

The first demonstration of CNP synthesis was performed by Calvo et al. who focused on the ratio of chitosan:TPP as a control of particle size and dispersity [33]. However, this widely used method is prone to uncontrollable intra- and intermolecular crosslinking between chitosan and TPP, resulting in aggregation, excess growth, and high polydispersity of CNPs [34,35,36]. 

The size and dispersity (reflected in PDI) of CNPs formation can be influenced by many processing factors, such as concentrations of chitosan and TPP, ionic strength, pH, reaction time, temperature, homogenization speed and duration, purification methods, etc. Kunjachan et al. found that chitosan and TPP concentrations had significant effects on particle size and PDI, while homogenization time only influenced the PDI [23]. However, Zu et al. observed that a time of 1–3 min decreased particle size, while 3–9 min introduced mechanical energy that necessitated higher particle density, thereby lowering specific surface area and increasing particle size [37]. Budi et al. (2020) observed a reduced CNPs size with lower TPP concentration due to a decrease in cross-link bonding and the formation of aggregate particles [38].

Sreekumar et al. reported that size of the CNPs is strongly dependent on the initial chitosan concentration, degree of chitosan acetylation, molecular weight, and salt concentration in the medium [39]. Using initial chitosan 0.1–5 mg/mL, 20–50% acetylation, 125–450 kDa, and NaCl 50–150 mM, CNPs with size 100–1200 nm and PDI 0.1–0.4 were produced [39]. Many of these experimental parameters were comparable to our study, but smaller size CNPs were achieved with our protocol. 

Due to limited resources, it was not feasible to investigate the influence of many processing factors on the size of CNPs formation. Thus, we had focused on assessing the influence of ionic strength, pH and purification methods on CNPs size and dispersity. These are described blow.

#### 2.1.1. Characteristics of Chitosan Nanoparticles under Ionic Strength Control

**Figure 3:**
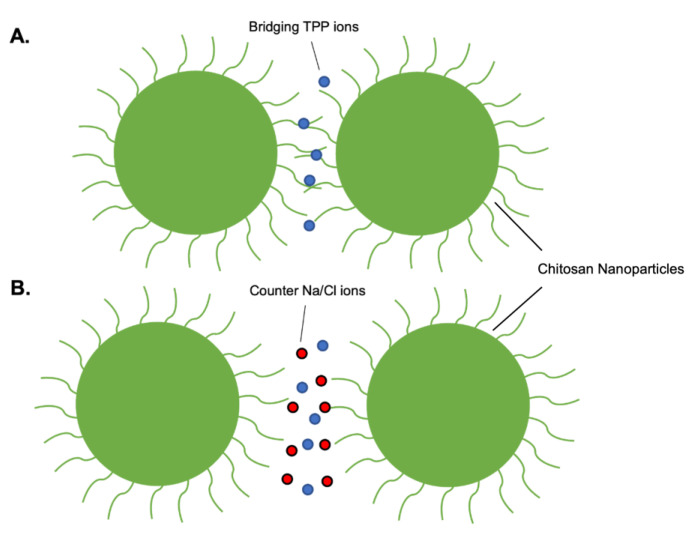
Effect of ionic strength on colloidal stability. (**A**) Bridging of chitosan nanoparticles by tripolyphosphate interactions with chitosan chains. (**B**) Disruption of tripolyphosphate-chitosan interactions by the presence of counter ions.

**Figure 4:**
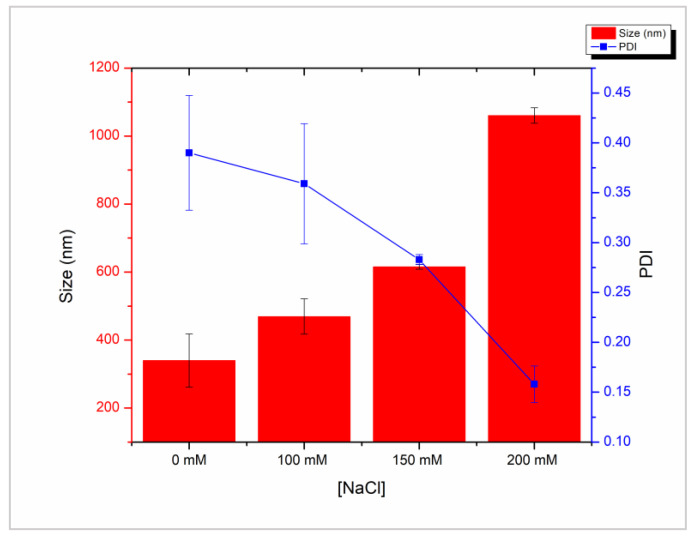
Size (red bars) and polydispersity index (blue line) of chitosan nanoparticles prepared with 1 mg/mL LMW chitosan and 1 mg/mL tripolyphosphate with increasing concentration of NaCl (0–200 mM). Measurements performed with dynamic light scattering in triplicate.

Control of CNPs size by altering ionic strength has been reported previously [24,40,41]. Increased ionic strength is proposed to limit electrostatic interactions between chitosan and TPP resulting in smaller monodisperse CNPs [24]. In particular, Cl^-^ ions compete with TPP and reduce bridging between CNPs, thereby increasing colloidal stability [40] (Figure 3). Although, at high ionic strengths, this effect will be offset by increased particle collisions induced by electrostatic screening. We found that the addition of increasing concentrations of NaCl (0–200 mM) correlated with a decrease in polydispersity index (PDI) of the CNPs, yet expectedly increased their size into the micro range (Figure 4).

At 100, 150 and 200 mM NaCl, PDI values decreased from 0.38 to 0.36, 0.27, and 0.14 respectively (Figure 4). This is in agreement with previous reports that found a decrease in PDI at all NaCl concentrations between 100 and 500 mM [24]. Likewise, Jonassen et al., found narrower CNPs size distributions with increasing ionic strengths [41]. We also found that particle size dramatically increased at higher concentrations, reaching >1000 nm at 200 mM NaCl (Figure 4). A similar phenomenon has been reported previously and is attributed to the screening of electrostatic repulsion, while the low PDI values are attributed to slower kinetics at high salt concentrations, thereby leading to a large population of monodisperse CNPs overtime [24]. 

#### 2.1.2. Characteristics of Chitosan Nanoparticles under pH Control

Alteration of pH has also been proposed as an effective means of avoiding aggregation and minimizing particle growth [25]. The kinetics of CNP formation are strongly governed by the protonation state of chitosan amine groups. Increasing the pH of the chitosan solution nearer to the amine group’s pKb value of 6.5 increases the number of internal hydrogen bonds, which restricts crosslinking with TPP ions. Consequently, the chitosan chains become more condensed within the particle, leading to a decrease in size, while the reduced availability of protonated amines do not allow for aggregation via bridging TPP ions. 

The pH of the TPP solution has been shown to influence CNP size and polydispersity [25,42,43]. Mattu et al. reported that CNPs prepared with basic TPP solutions (pH 9.5) had a larger size compared with those obtained with acidic TPP solutions (pH 5.5). It is proposed that lower pH values limit TPP interaction with chitosan, due to the presence of H_3_O^+^ and H^+^ counter ions. At TPP pH >5, both the size and PDI of CNPs has been reported to increase dramatically [25], therefore, we examined the pH effect by fixing the chitosan solution to pH 5 and altering the TPP solution pH between 2 to 4.

**Figure 5:**
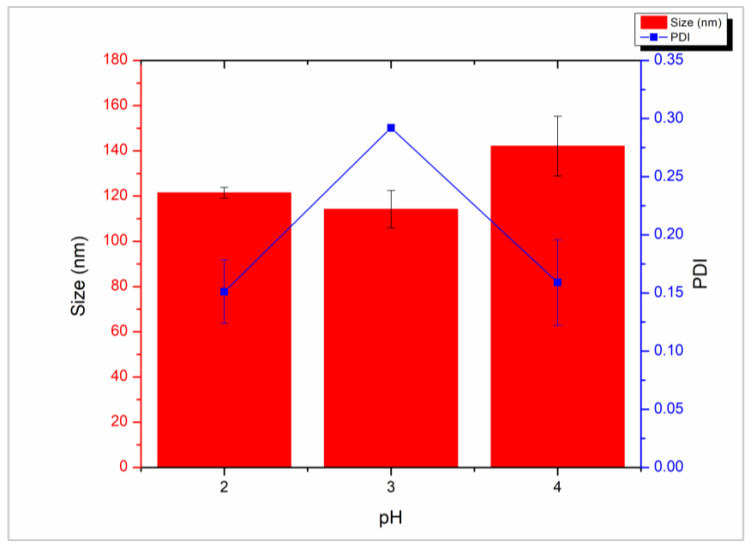
Size (red bars) and polydispersity index (blue line) of chitosan nanoparticles prepared with 1 mg/mL LMW chitosan and 1 mg/mL tripolyphosphate with increasing tripolyphosphate solution pH (2–4). Measurements performed with dynamic light scattering in triplicate.

Consistent with previous findings, we also observed that smaller CNPs were formed at lower TPP pH [25,42,43]. CNP size was the largest at pH 4, with a size of 142 nm, compared to 117 nm, and 121 nm for pH 3 and 2 respectively (Figure 5). PDI values fluctuated with varying pH but were lowest at 0.15 for pH 2 (Figure 5). These changes are minor and suggest that in this TPP pH range, the pH of the chitosan solution is the determining factor for particle growth and polydispersity. Therefore, altering the pH of the TPP solution may be used for fine-tuning of size and PDI after an appropriate pH for the chitosan solution is found.

Our experimental results are in agreement with previous reports [25], and the method we used can serve as a more efficient control for particle size and dispersity than ionic strength. However, our desired goal of <100 nm CNPs could not be achieved through this control alone. 

#### 2.1.3. Characteristics of Chitosan Nanoparticles with a Homogenizer

In order to synthesize smaller CNPs, an alternate homogenization method was investigated. A high number of procedures in the literature employ magnetic stirring throughout the growth phase and rely on stir speed and time to control for particle size and polydispersity. This is not a new idea, as the increased shear forces introduced at higher speeds disrupts particle formation, resulting in smaller sizes. This idea is commonly used in the sol-gel technique, for formulating silica NPs of a desired size. 

We have found that the use of magnetic stirring is unable to formulate <100 nm CNPs with low dispersity, even when chitosan and TPP ratios are selected appropriately, or other factors such as ionic strength and pH are controlled, as discussed previously. However, implementing rigorous homogenization with a handheld homogenizer motor at 7000 rpm for 2 min consistently resulted in <80 nm CNPs. The ability to formulate small CNPs with low polydispersity, without the use of additional reagents and timely procedures, is an important step in developing this efficient synthesis procedure. Similar resulted have been reported in which applying higher homogenization speeds (1000 to 7000 rpm) decreased particle size [44]. However, our protocol is the first to achieve sub-100 nm sizes using this mixing method.

### 2.2. Purification and Collection of Chitosan Nanoparticles

#### 2.2.1. Purification of Chitosan Nanoparticles with Ultracentrifugation

The next barrier to overcome in this procedure was the collection of CNPs from the homogenized solution. The small size of the CNPs restricted our ability to wash and collect via centrifugation, a staple method in the purification step of most NP syntheses. Ultracentrifugation is necessary for the isolation of particles <100 nm, however, the high centrifugal forces consequently disrupted the structure of the CNPs, resulting in a rigid pellet that was not susceptible to resuspension.

Attempts to resuspend the pellet via vortexing and bath sonication resulted in a slightly cloudy solution with visible aggregates from the pellet. The measured size and PDI of this sample were 219 nm and 0.7 respectively. These DLS results suggest that any CNPs that had resuspended had lost their original structure and uniformity. Decreases to ultracentrifugation speed (149,000 RCF to 118,000 RCF) and time (60 min to 40 min) were assessed, however similar results were observed.

#### 2.2.2. Purification of Chitosan Nanoparticles with Size Exclusion Chromatography

Size exclusion chromatography was evaluated next as a means to separate the CNPs from any precursor material retained in solution, while maintaining their size and uniformity. However, attempts to purify in this manner presented other challenges.

**Figure 6:**
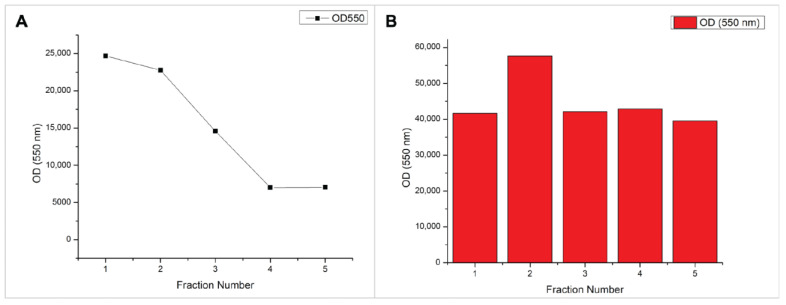
Light scattering results of chitosan fractions collected with size exclusion chromatography. (**A**) Chitosan nanoparticles synthesized under homogenization. (**B**) LMW chitosan dissolved in 1% acetic acid to a concentration of 1 mg/mL.

Light scattering and DLS measurements of initial samples suggested that our CNPs of interest primarily eluted over two fractions, while other fractions contribute to a polydisperse sample (Figure 6A, Table 1). The collected fractions were pooled and subjected to a second pass through the column. In comparison to the scattering profile of CNPs, unlinked chitosan polymers display similar scattering values across all 5 eluted fractions, with the exception of fraction 2 (Figure 6B). Furthermore, these scattering values are significantly larger than those recorded for the CNPs (OD 550 nm: ~40,000 to ~24,000). This supports our assumption that CNPs are being collected and that little unlinked chitosan polymer remains after particle formation.

The resulting fractions expected to contain CNPs of the desired size range were pooled and measured using DLS, while the subsequent fractions were pooled separately (Table 1). Unfortunately, this process of pooling fractions to avoid high polydispersity greatly diluted the CNPs. This in turn makes DLS measurements unreliable, and even more importantly, limits the use of these CNPs for relevant applications. 

Table 1 summarizes the sizes of CNPs in fractions eluted with size exclusion chromatography (SEC). The initial exclusion may be used to obtain a certain number of particles in this range. However, this method may not be efficient as evident in the changes between Z-average as well as in the major species present. Ultimately, this method diluted CNP concentrations to a point where their characterization became unreliable. 

This method of purification greatly limits the use for CNP synthesis on large scales, where high concentrations and monodispersity are required.

#### 2.2.3. Purification of Chitosan Nanoparticles Using Syringe Filtration

To effectively purify CNPs out of the homogenized solution and maintain high concentration values and monodispersity, we opted to filter the solution through a 0.1 μm polyethersulfone filter. This would remove any larger particles from solution that were not within the desired size range, increasing monodispersity.

This method proved the most effective of the three for collection of <80 nm CNPs with relatively low PDI values of ~0.3 (Table 2). Following homogenization, CNPs are drawn into a syringe, which is then loaded into a syringe pump (Harvard Instruments). CNPs are then infused through the filter into a collection vial at a rate of 0.15 mL/min. Large nanoparticles and unreacted chitosan may cause clogging of the filter, and the infusion can be paused to facilitate filter replacement. 

The above procedure was replicated four times and samples were measured across two facilities with different characterization instruments, including DLS at University of Calgary and Tunable Resistive Pulse Sensing (TRPS) with Exoid-034 at IZON Science (Christchurch, New Zealand). Results were strikingly similar. 

Zeta potential measurements of these CNPs revealed a value of ~+20 mV. This is an indication of adequate particle stability, as electrostatic repulsion between one another will prevent aggregation over time.

**Figure 7:**
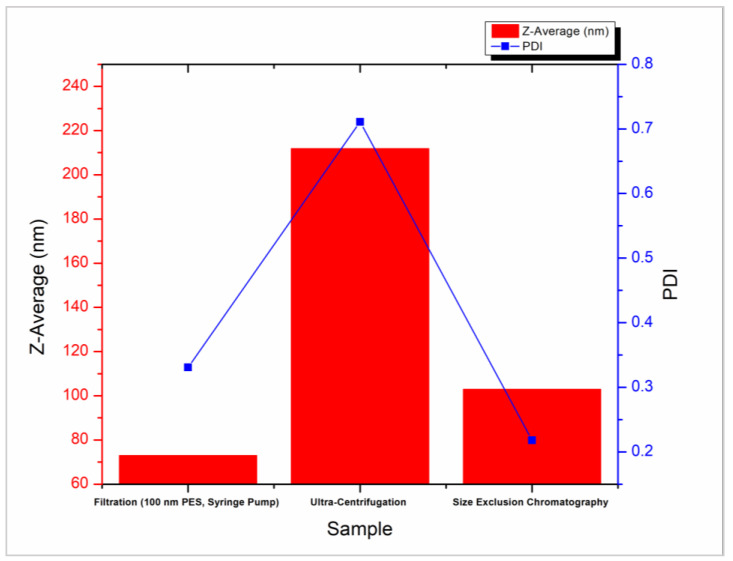
Summary of three chitosan nanoparticle purification methods and their ability to isolate nanoparticles of small size and low polydispersity.

Considering the three approaches used in this study to purify CNPs following their synthesis using homogenization, purification by syringe filtration has proved to be the most effective way to isolate these nanoparticles within the <80 nm size range and to have adequate monodispersity (Figure 7). Ultracentrifugation applies significant force resulting in the formation of gels, while SEC may be capable of isolating for particle size the wide elution range and indirect dilution of samples introduces unreliability into measurements and hampers scalability for application.

### 2.3. Characterization of Chitosan Nanoparticles with Scanning Electron Microscopy

**Figure 8:**
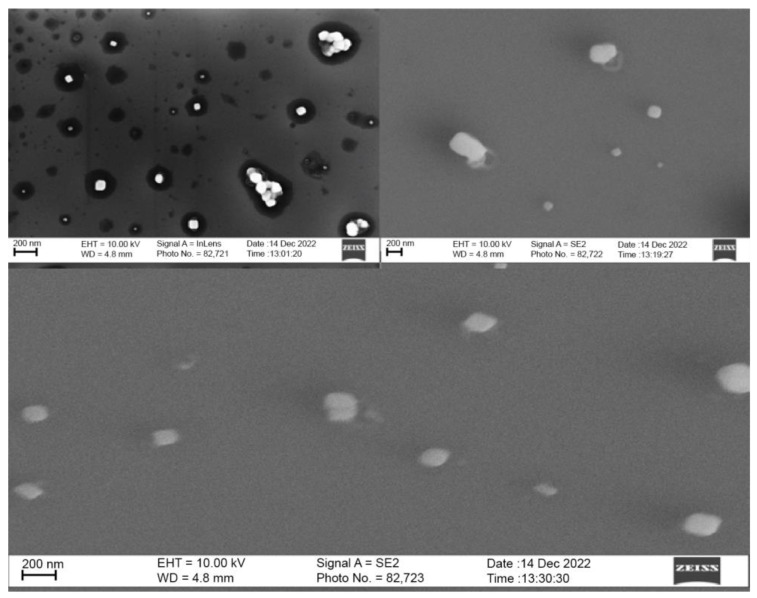
SEM characterization of CNPs following synthesis using homogenization and purification using filtration. Images obtained using both InLens and SE2 Signal A (Zeiss ∑igma VP Scanning Electron Microscope).

CNPs were analyzed via SEM as a secondary size characterization method in addition to DLS. SEM images were taken for CNPs synthesized with a homogenizer and purified with syringe filtration, as this procedure proved effective for our goals of formulating <100 nm CNPs with low polydispersity. The CNPs displayed a circular morphology, similar to literature, and were split into two size populations, with average sizes of 75 ± 8 nm and 33 ± 11 nm (Figure 8).

The larger population is in strong agreement with DLS measurements, while the presence of a second smaller population is reflective of the somewhat poor PDI value (Table 2). These smaller particles may be the result of an incomplete growth phase. Therefore, increasing the homogenization time may allow for these particles to equilibrate with the average size. 

## 3. Materials and Methods

### 3.1. Materials

Low molecular weight chitosan (MW 50–190 kDa, degree of deacetylation 75–85%), sodium tripolyphosphate, and sodium chloride were purchased from Sigma Aldrich (Markham, ON, Canada). Acetic acid was obtained from EMD chemicals (Mississauga, ON, Canada). 

### 3.2. Synthesis of Chitosan Nanoparticles

**Figure 9:**
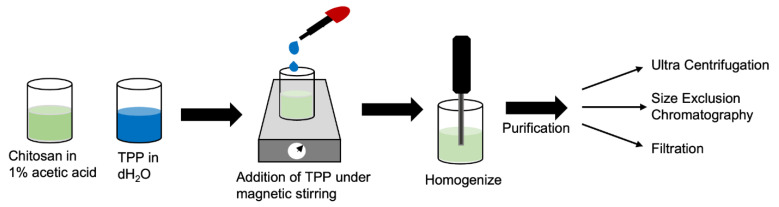
Schematic of chitosan nanoparticle synthesis.

The procedure was adapted from Iswanti et al. [45]. Briefly, the chitosan solution was prepared by dissolving chitosan in 1% acetic acid to a concentration of 1 mg/mL. The chitosan solution (21 mL) was set on a magnetic stir plate (1000 rpm) and 9 mL of 1 mg/mL aqueous TPP solution was added dropwise at a rate of 1 mL/min. The mixture was homogenized at 7000 rpm over 2 min with a handheld homogenizer motor (Fisherbrand^TM^ 150) prior to purification and collection (Figure 9). 

### 3.3. Purification of Chitosan Nanoparticles

CNPs were washed and collected using three different methods: (1) ultracentrifugation and resuspension in a 1% acetic acid solution; (2) size exclusion chromatography and pooling of collected fractions; and (3) filtration through a 0.1 μm polyethersulfone filter.

Ultracentrifugation was carried out with an Optima L-1000K centrifuge (Beckman Coulter, Indianapolis, IN, USA) at 149,000 RCF for 60 min (18 °C). The pellet was resuspended in 1% acetic acid (10 mL) via vortexing and bath sonication. Alternatively, CNPs were added to a Sephadex G25 size exclusion column. Scattering spectra (500 nm excitation and emission wavelengths) were recorded with a spectrofluorometer (Shimadzu, Columbia, MD, USA) for collected fractions (10 drops/fraction) to determine the elution profile of CNPs. Lastly, the CNP suspension was filtered through a 0.1 µm PES syringe filter with a syringe pump (Harvard Apparatus, Holliston, MA, USA) at a flow rate of 0.3 mL/min. 

### 3.4. Synthesis of Chitosan Nanoparticles with Ionic Strength Control

Adapting methods by Sawtarie et al. the chitosan and TPP solutions were prepared to a concentration of 2 mg/mL in 6 mL of 2% acetic and 2 mL of ddH_2_O, respectively [24]. Three NaCl solutions were made to concentrations of 200, 300, and 400 mM. The NaCl solutions were then mixed with the chitosan and TPP solutions at a 1:1 volume ratio to bring the final precursor concentrations to 1 mg/mL and final NaCl concentrations to 100, 150, and 200 mM. CNPs were then synthesized by dropwise addition of the TPP-NaCl solution (4 mL; 1 mL/min) to the chitosan-NaCl (12 mL) solution set on a magnetic stir plate at 1000 rpm. After 5 min, the CNP suspension was subject to 2 rounds of microcentrifugation (18,000 RCF, 40 min) and resuspended in 10 mL ddH_2_O.

### 3.5. Synthesis of Chitosan Nanoparticles with pH Control

Adapting methods by Masarudin et al. the chitosan and TPP solutions were prepared to a concentration of 1 mg/mL in 12 mL of 1% acetic and 4 mL of ddH_2_O respectively [25]. The chitosan solution was adjusted to pH 5 with 1 M HCl and three TPP solutions were adjusted to pH values of 2, 3, and 4 with 1 M HCl. CNP synthesis was carried out by dropwise addition of the TPP solution (1 mL/min) to the chitosan solution under magnetic stirring at 1000 rpm. After 5 min of homogenization, the CNPs were collected via centrifugation (18,000 RCF, 40 min) and resuspended in 2 mL ddH_2_O.

### 3.6. Characterization of Chitosan Nanoparticles

The concentration of CNP samples was determined by drying and weighing of a 50 µL aliquot. The size, PDI, and zeta potential of CNPs were measured using a Zetasizer Nano ZS (Malvern, Worcestershire, UK). Measurements were performed in triplicate at 25 °C with 10 runs per measurement and 10 s per run. Size and PDI of CNPs were measured in disposable polystyrene cuvettes and zeta potential was measured in folded capillary zeta cells.

Imaging of the CNPs was done with a scanning electron microscope (Zeiss Sigma VP, Field Emission, Zeiss, Heidenheim, Germany) using an InLens and SE2 Signal A. Samples (50 µL) were deposited on silicon wafers and dried under nitrogen prior to imaging.

## 4. Conclusions and Future Work

Synthesis methods of CNPs are many, however, reproducible methods for achieving sub-100 nm particles are lacking in the literature. Certain groups employ controls on salt concentration and pH to facilitate the growth phase in producing these desired sizes, however, these methods can be time consuming and costly for large-scale productions. Our approach utilizes rigorous homogenization followed by filtration of the CNP solution and requires no additional reagents, which greatly simplifies the synthesis procedure. We have demonstrated reproducible results in synthesis of high yield, monodispersed, 68–77 nm CNPs.

The positively charged sub-100 nm CNPs could readily interact with negatively charge proteins and DNA, making them an ideal precursor for the development of DNA-labelled, protein-coated virus surrogates [30]. We do not anticipate any difficulty in encapsulating biomolecules within the sub-100 nm CNPs developed in this study. We had previously encapsulated DNA tracers within 310 nm chitosan microparticles with a thin alginate outer layer [46]; encapsulation changed very little on the size of chitosan microparticles. Furthermore, the amino groups present in chitosan enable CNPs to be easily functionalized, leading to many potential applications such as water and wastewater treatment, food-packaging, biomedical and agricultural products.

## Figures and Tables

**Table 1:** Results of Size Exclusion Chromatography of CNPs synthesized using 1 mg/mL chitosan and 1 mg/mL tripolyphosphate.

SEC Fraction	Major Species Hydrodynamic Diameter (nm)	Z-Average (nm)
Fraction 1	62.12	146.7
Fraction 2	75.42	60.15
**SEC Pooled Fractions**	**Z-Average (nm)**	**PDI**
1, 2 Pooled	103	0.218
3, 4, 5, 6, 7 Pooled	95.18	0.399
Not Subjected	83.36	0.301

**Table 2:** Characteristics of chitosan nanoparticles synthesized with a 1 mg/mL chitosan solution and 1 mg/mL tripolyphosphate solution and subsequently purified using syringe filtration.

Facility/Equipment	Size (nm)	PDI	Zeta Potential (mV)	Particle Concentration (Particle/mL)
University of Calgary/Malvern ZetaSizer Nano ZS	73	0.344	10.17 ± 3.35	-
University of Calgary/Malvern ZetaSizer Nano ZS	68	0.330	21.00 ± 0.70	-
University of Calgary/Malvern ZetaSizer Nano ZS	77	0.319	21.10 ± 1.98	-
IZON Science (Christchurch, New Zealand) TRPS with Exoid	72	0.227 *	-	3.57 × 10^9^

* calculated from the square of the standard deviation divided by the mean particle diameter.

## Data Availability

Data will be made available upon request.
